# Implementation research approaches to promoting universal health coverage in Africa: a scoping review

**DOI:** 10.1186/s12913-021-06449-6

**Published:** 2021-05-03

**Authors:** Chukwudi A. Nnaji, Charles S. Wiysonge, Joseph C. Okeibunor, Thobile Malinga, Abdu A. Adamu, Prosper Tumusiime, Humphrey Karamagi

**Affiliations:** 1 Cochrane South Africa, South African Medical Research Council, Cape Town, South Africa; 2 School of Public Health and Family Medicine, University of Cape Town, Cape Town, South Africa; 3 Department of Global Health, Stellenbosch University, Cape Town, South Africa; 4 Health Systems and Services Cluster, World Health Organization Regional Office for Africa, Brazzaville, Congo

**Keywords:** Implementation research, Evidence, Universal health coverage, Access, Equity

## Abstract

**Background:**

Implementation research has emerged as part of evidence-based decision-making efforts to plug current gaps in the translation of research evidence into health policy and practice. While there has been a growing number of initiatives promoting the uptake of implementation research in Africa, its role and effectiveness remain unclear, particularly in the context of universal health coverage (UHC). Hence, this scoping review aimed to identify and characterise the use of implementation research initiatives for assessing UHC-related interventions or programmes in Africa.

**Methods:**

The review protocol was developed based on the methodological framework proposed by Arksey and O’Malley, as enhanced by the Joanna Briggs Institute. The review is reported in accordance with the Preferred Reporting Items for Systematic Reviews and Meta-Analyses Extension for Scoping Reviews (PRISMA-ScR). MEDLINE, Scopus and the Cochrane Library were searched. The search also included a hand search of relevant grey literature and reference lists. Literature sources involving the application of implementation research in the context of UHC in Africa were eligible for inclusion.

**Results:**

The database search yielded 2153 records. We identified 12 additional records from hand search of reference lists. After the removal of duplicates, we had 2051 unique records, of which 26 studies were included in the review. Implementation research was used within ten distinct UHC-related contexts, including HIV; maternal and child health; voluntary male medical circumcision; healthcare financing; immunisation; healthcare data quality; malaria diagnosis; primary healthcare quality improvement; surgery and typhoid fever control. The consolidated framework for implementation research (CFIR) was the most frequently used framework. Qualitative and mixed-methods study designs were the commonest methods used. Implementation research was mostly used to guide post-implementation evaluation of health programmes and the contextualisation of findings to improve future implementation outcomes. The most commonly reported contextual facilitators were political support, funding, sustained collaboration and effective programme leadership. Reported barriers included inadequate human and other resources; lack of incentives; perception of implementation as additional work burden; and socio-cultural barriers.

**Conclusions:**

This review demonstrates that implementation research can be used to achieve UHC-related outcomes in Africa. It has identified important facilitators and barriers to the use of implementation research for promoting UHC in the region.

**Supplementary Information:**

The online version contains supplementary material available at 10.1186/s12913-021-06449-6.

## Background

The need for health decision making to be informed by empirical evidence has been identified as a vital step for achieving universal health coverage (UHC) and equitable access to high quality health care [1, 2]. It has been recognised that decisions informed by research evidence have the potential to promote equitable service delivery and improve health outcomes at population level, while strengthening health systems [2]. The World Health Organization (WHO) defines UHC as “ensuring that all people have access to needed health services (including prevention, promotion, treatment, rehabilitation and palliation) of sufficient quality to be effective while also ensuring that the use of these services does not expose the user to financial hardship” [3]. Since the 1978 Alma-Ata Declaration and the 1986 Ottawa Charter for Health Promotion, the right to the highest attainable standard of physical and mental health has gained increasing attention [4]. As a result of this prioritisation, UHC was adopted as a target of the Sustainable Development Goals (SDG), with the aspiration that countries will achieve this by 2030 [5].

With the increasing momentum of global efforts towards the attainment of UHC, countries are often faced with difficult choices regarding the most effective use of available health resources, particularly in contexts of resource limitation, competing healthcare needs and political priorities [6]. Given this inherent complexity, UHC decision making requires adequate consideration of best available and contextually applicable research evidence [6, 7]. While investment in health research and research outputs have grown considerably in Africa over the years, there remain enormous gaps in translating available research evidence into health policy and practice [8]. This so-called ‘know–do gap’ has resulted in suboptimal gains from allocated health resources, in spite of growing investment towards the actualisation of UHC in Africa [2, 9]. The gap is accentuated by the region’s high burden of communicable and non-communicable diseases [10, 11].

Implementation science has emerged in response to this critical gap [12]. Implementation science is an integral part of the broader Evidence-informed Decision Making (EIDM) enterprise. EIDM involves processes of distilling and disseminating the best available evidence from research, practice and experience and using that evidence to inform and improve public health policy and practice [13, 14]. Knowledge translation, knowledge transfer and translational research are EIDM concepts that are closely related to implementation science, used to refer to the processes of moving research-based evidence into policy and practice, through the synthesis, dissemination, exchange and application of knowledge to improve the health of the population [13, 15–17]. Although there may be nuanced differences in their conceptualisation, these terms essentially have similar goals and practical implications for improving health outconmes [15–17].

There has been no clear consensus on the definition of implementation science [18]. In 2015, Odeny and colleagues published a review of the literature that found 73 unique definitions [19]. Broadly, implementation science has been defined as “the scientific study of methods to promote the systematic uptake of research findings and other evidence-based practices into routine practice, and, hence, to improve the quality and effectiveness of health services.” [16] Since the field of implementation science has cogent applications for both clinical and public health settings, this definition is more encompassing and highlights the field’s broad nature. The process of inquiry in implementation science is through research, which builds on traditional scientific methods, but focuses on a unique set of questions to improve the use of research in implementation [16, 19]. Thus, implementation science offers the toolkit for addressing the know-do gap [16, 20, 21].

In 2006, Eccles and Mittman proposed a working definition for the emerging field of implementation research – defining it as the “scientific study of methods to promote the adoption and integration of evidence based practices, interventions and policies into routine health care and public health settings.” [21] More recently in 2013, the WHO’s Alliance for Health Policy and Systems Research (AHPSR) defines it as “the scientific study of the processes used in the implementation of initiatives as well as the contextual factors that affect these processes.” [18] This definition highlights a defining feature of implementation research; that is, going beyond the study of methods of promoting the uptake of evidence into routine practice, to studying the contextual facilitators and barriers to evidence-based implementation [17, 18]. For this reason, implementation research has been regarded as the heart and soul of implementation science [17]. While implementation science and implementation research have been interchangeably used in literature, implementation research is the reference term for this review.

The role of implementation research encompasses health policy development, policy communication, as well as programme planning, implementation and evaluation [17, 18]. Various conceptual theories and frameworks have been used to guide implementation research efforts across diverse settings. Some of the most commonly used frameworks include the Consolidated Framework for Implementation Research (CFIR), Reach Effectiveness Adoption Implementation Maintenance (RE-AIM) and Theoretical Domains Frameworks (TDF) [22, 23]. To facilitate the use of implementation research in health system decision making and routine practice, there have to be: (a) availability of rigorous, robust, relevant, and reliable evidence, (b) decision-makers’ appreciation of the value and importance of empirical evidence in decision making processes (c) a trusting, mutually respectful and enduring engagement between evidence producers and decision makers [6, 13, 24].

Various implementation research initiatives and efforts for improving health outcomes have emerged in the African region in the last decade [13, 17, 25–28]. In spite of this substantial growth, implementation research uptake, effectiveness and scale-up in the region is challenged by numerous barriers [25–27]. These include inadequate research funding; limited availability and access to research training opportunities and paucity of contextually relevant implementation research models [27]. Another major barrier is the lack of political will or commitment to health-related implementation research and the broader UHC agenda, the pursuits of which are intrinsically political and cannot be attained without adequate political support [27, 29]. Other reported barriers include the untimeliness of research and, of course, fragile collaboration between researchers and users of evidence like policy-makers and frontline programme implementers [2, 7, 30, 31].

### Study rationale

Globally, evidence-based health decision making and implementation models are being adopted as approaches for improving the health of populations [7, 16, 32]. While there has been a growing number of institutions and initiatives promoting the uptake of implementation science and implementation research in Africa, the characteristics and role of these initiatives remain unclear [33, 34].

There is a dearth of literature on synthesised bodies of evidence on the role of implementation research in Africa’s health systems and the extent to which it has been used in the context of UHC on the continent. With limited funding and institutional research capacity to drive implementation research efforts in Africa, there is an urgent need to seek out cross-country learning opportunities that can bolster understanding of implementation research and broader EIDM strategies in the region [11, 35]. A better understanding is important to stimulate greater synergy and collaboration between evidence producers and users, while optimising the overall impact of implemented programmes and health systems strengthening in the region.

Scoping reviews represent an appropriate methodology for thematically reviewing large bodies of literature in order to generate an overview of existing knowledge and practice, as well as identifying existing evidence gaps [36, 37]. Like full systematic reviews, scoping reviews employ methods that are transparent and reproducible, using pre-defined search strategies and inclusion criteria [38, 39]. However, unlike systematic reviews which often target specific and narrow research questions, scoping reviews typically have a broader focus – including the nature, volume and characteristics of the literature in order to identify, describe and categorise available evidence on the topic of interest [37–39].

Therefore, this scoping review seeks to fill existing gaps in the availability of synthesised evidence on implementation research in the context of UHC, health equity and health systems strengthening within the African region. It maps the region’s implementation research strategies, major actors, reported outcomes, facilitators, and barriers from a diverse body of literature. Ultimately, it seeks to provide a holistic and user-friendly evidence summary of implementation research and key issues in the region for researchers, policymakers and implementers, while identifying lingering knowledge and practice gaps to inform future implementation research efforts.

## Methods

### Protocol design

An a priori protocol for this review, which has been published elsewhere [40], was designed in accordance with the Arksey and O’Malley scoping review methodology [41], as enhanced by the Joanna Briggs Institute (JBI) [42]. The JBI’s enhanced framework expands the six stages of Arksey and O’Malley into 9 distinct stages for undertaking a scoping review: (1) defining the research question; (2) developing the inclusion and exclusion criteria; (3) describing the search strategy; (4) searching for the evidence; (5) selecting the evidence; (6) extracting the evidence; (7) charting the evidence; (8) summarising and reporting the evidence and (9) consulting with relevant stakeholders. The protocol was disseminated throughout the extensive professional networks of the author group and the World Health Organization (WHO) to solicit feedback. Findings of the review are reported using the Preferred Reporting Items for Systematic reviews and Meta-Analyses extension for Scoping Reviews (PRISMA-ScR) checklist [43].

### Conceptual framework

**Fig. 1:**
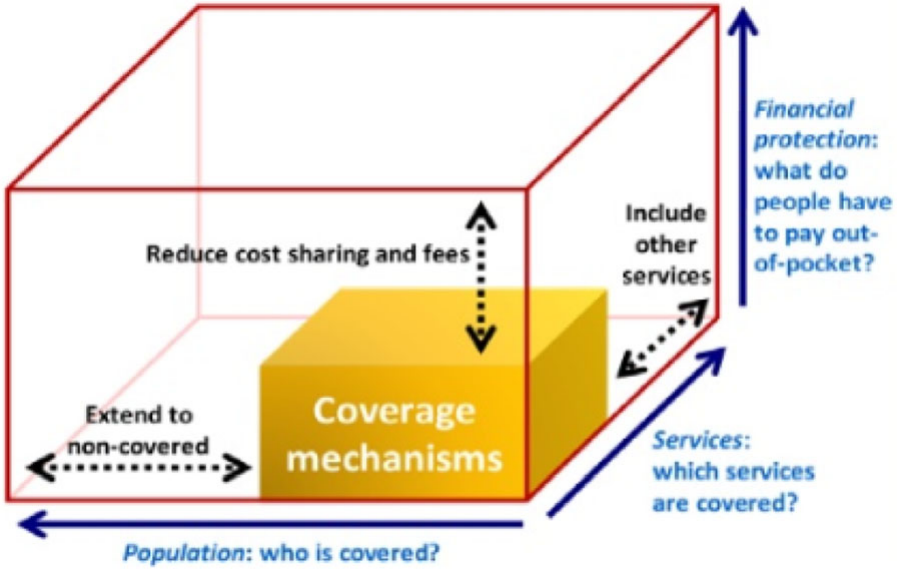
The World Health Organization’s Universal Health Coverage (UHC) Cube

This scoping review used the WHO’s UHC Cube conceptual framework for mapping the processes and outcomes between implementation research and UHC [44]. This framework uses a cube (see Fig. 1) to depict the multidimensional nature and outcomes of UHC. The cube illustrates three core dimensions of conceptualising UHC: population coverage of health-related social security systems, financial protection, and access to quality health care according to need [44, 45]. These dimensions provide an assessment framework for UHC-targeted initiatives, reflecting how many (or what proportion of) people received various needed health services of sufficient quality, while being protected from undue financial risks [44]. Although the framework does not take into account specific contextual factors, it has been widely used globally for conceptualising UHC across diverse health systems and contexts [45–47].

### Defining the research question

Through consultation with the research team and key stakeholders, the overall main research question was defined as: ‘What are the nature and scope of implementation research initiatives for improving equitable access to quality promotive, preventive, curative, rehabilitative and palliative health services in Africa?’ For the purpose of this review, implementation research has been defined within the broader frameworks of implementation science, knowledge translation and evidence informed decision making. Based on the primary research question, the following specific research questions were defined:

1. How has implementation research been used to assess or evaluate UHC-related interventions and programmes in the African Region?
2. What are the facilitators and barriers to the application, uptake and sustainability of implementation research in UHC-related contexts in Africa?

### Inclusion and exclusion criteria

#### Inclusion criteria

These were generated using the PCC (Population, Concept and Contexts) framework, proposed by Peters and colleagues [48]. This framework is more appropriate for scoping reviews, compared with the commonly used PICO (Population, Intervention, Comparator and Outcome) framework, as it allows for the consideration of publications that may not feature all of the four PICO elements (e.g. lacking an outcome or comparator/control). Eligible population included evidence producers (health researchers), intermediaries (such as knowledge brokers and implementation research institutions) and evidence users (such as health policymakers, programme implementers like non-government organisations and healthcare providers). There are two concepts of interest for this review, an intervention concept (implementation research) and an outcome concept (UHC). The two concepts of interest are implementation science and UHC. To be considered for inclusion, implementation research initiatives were any activity using a specified implementation research framework or theory design to facilitate the use of research in UHC-related planning, decision making and implementation. Studies with or without comparator between implementation research strategies and control were eligible for inclusion. Outcomes included health service coverage, access (service utilisation and quality of care) and financial risk protection, in line with the UHC Cube framework [44]. Studies that evaluated specific health programme implementation outcomes, barriers or facilitators were included, provided the implementation involved the use of specific implementation research approaches, frameworks or theories. Health systems in Africa were the context of interest. All primary study designs were eligible for inclusion. Further details about the eligibility criteria have been published elsewhere [40].

#### Exclusion criteria

Literature focused solely or mainly on theoretical and conceptual development of implementation research were excluded, as were those evaluating implementation research knowledge and practice outcomes without interventions, those evaluating implementation outcomes without using specific implementation research frameworks and those discussing implementation research strategies that are not UHC-related. Multinational literature involving African and non-African countries and meeting inclusion criteria were excluded if country-specific information could not be abstracted.

### Searching the evidence

The search strategy was developed and applied in accordance with the Peer Review of Electronic Search Strategies (PRESS) guidelines [49]. It was adapted for the different databases using appropriate controlled vocabulary and syntaxes. The search strategy used search terms that are sensitive enough to capture literature relevant to implementation research, with due cognisance of the field’s diverse and overlapping nomenclature and search filters for African countries. An initial exploration of current available literature on implementation research and UHC guided the selection of search terms, ensuring they are inclusive enough to capture any UHC-related implementation research intervention. Details of the search strategies for each database are outlined in Additional file 1.

A comprehensive literature search was conducted on the following electronic databases: MEDLINE (via PubMed), Scopus and Cochrane Library (including the Cochrane Central Register of Controlled Trials (CENTRAL) and the Database of Abstracts of Reviews of Effects (DARE)). Each database was searched from inception until August 15, 2020. Additionally, relevant grey literature was searched for implementation research-related reports, including the website of the WHO Alliance for Health Policy and Systems Research (AHPSR). Websites of known implementation research institutions, networks and collaborations were explored. We also conducted a hand-search of reference lists of relevant literature to identify for potentially eligible literature. No language restriction was applied. We planned for translation if a potentially eligible literature was published in a language other than English. Further details of the planned search strategies are described in the published review protocol [40].

### Selecting the evidence

The review process consisted of two levels of screening: a title and abstract screening to identify potentially eligible publications and review of full texts to select those to be included in the review based on pre-defined inclusion/exclusion criteria. For the first level of screening, titles, and abstracts of all retrieved citations from the search output were screened. Articles that were deemed relevant were included in the full-text review. In the second step, the retrieved full texts were assessed to determine if they met the inclusion/exclusion criteria.

### Extracting the evidence

A pre-tested data extraction tool was used to extract relevant info from included literature. Extracted information included study characteristics (author, year of publication and country context), study design, implementation research details (platform, framework, strategies and target participants), UHC-related target outcomes as well as identified contextual facilitators and barriers. All extracted data were validated with the full texts before analysis.

## Results

**Fig. 2:**
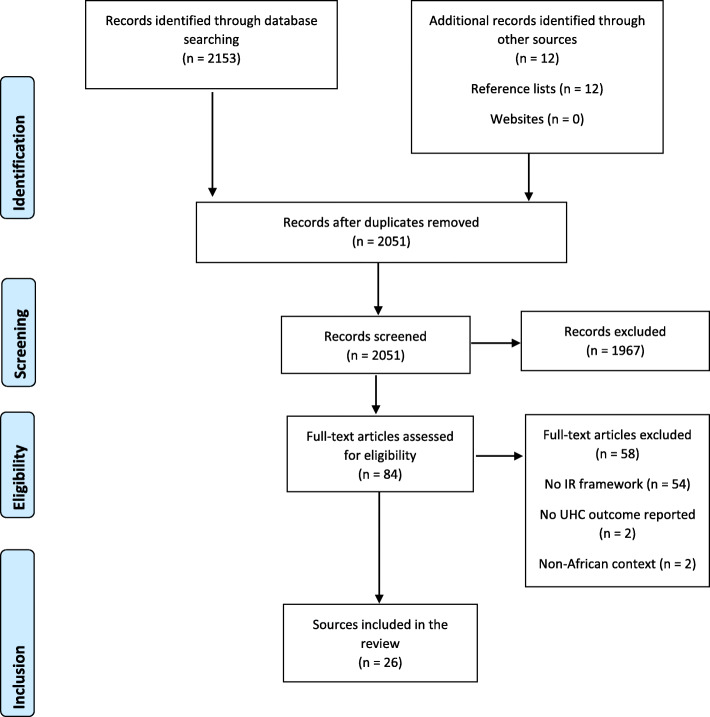
PRISMA flow diagram showing the study selection process

Our database search yielded 2153 records. We identified 12 additional records from hand search of reference lists. After removal of duplicates, we had 2051 unique records. The titles and abstracts of these articles were screened, of which 1967 clearly ineligible records were excluded. We conducted a full-text review of the remaining 84 articles, of which 58 were excluded: 54 were removed due to citing but not utilising any implementation research framework, two did not report UHC-related outcomes and another two were not based in an African context. Figure 2 describes the study selection process.

We included 26 studies in the review (see Table 1: summary of characteristics of included studies). Findings were reported using the Preferred Reporting Items for Systematic reviews and Meta-Analyses extension for Scoping Reviews (PRISMA-ScR) checklist [43], and summarised narratively based on identified themes.

**Table 1:** Summary of characteristics of included studies

Study	Country	IR Framework	Study design	IR approach	Target actors/participants	Reported facilitators/barriers	Targeted UHC outcomes
Adamu 2019 [50]	Nigeria	TDF	Qualitative	Pre-implementation use of IR to guide the implementation of a quality improvement programme for improving routine childhood immunisation services	Parents and caregivers of children attending primary health care facilities	**Facilitators:** Perceived benefits of vaccines and adequate communication of benefits **Barriers:** inadequate knowledge of the vaccines, non-screening of home-based records, health worker’s refusal to offer immunization services, and husband’s refusal due to socio-cultural beliefs	To reduce missed opportunities for vaccination and improve routine childhood immunisation coverage
Adamu 2020 [51]	Nigeria	CFIR	Mixed methods	IR used to guide data collection, data analysis and post-implementation evaluation of routine childhood immunisation services	Primary health care facility staff	**Facilitators:** Intervention flexibility, self-efficacy among health workers, health workers’ confidence in the intervention, services integration. **Barriers:** Vaccine stock out, faulty cold chain infrastructure, lack of incentives, and socio-cultural beliefs	Improved access to routine childhood immunisation services
Anaba 2019 [52]	Ghana	NPT	Quantitative	IR used to guide data collection, data analysis and post-implementation evaluation of malaria rapid diagnostic test (mRDT) practices among health workers	Health workers	**Facilitators:** three or more years of experience, clarity on the benefits, availability of innovation champions or initiators and readiness for change practices **Barriers:** poor monitoring	Optimised intention to use mRDT in the diagnosis of malaria
Barac 2018 [53]	Multi-country, including Nigeria and South Africa	CFIR	Mixed methods	IR used to guide data collection, data analysis and post-implementation evaluation of typhoid control interventions	Organisations involved in implementation; Health policy and health system leaders at national or subnational levels	**Facilitators:** Use of multiple implementation strategies to target behaviour change. **Barriers:** Limited resources and planning, habitual behaviours and cultural practices.	Better understanding of the effectiveness of typhoid control interventions
Bardosh 2017 [54]	Multi-country, including Kenya	CFIR	Qualitative	IR-oriented mid-implementation and evaluation of a mobile health (mHealth) intervention.	Health care workers, research team members, and community members	**Facilitators**: Perceived positive impact on patients **Barriers:** Illiteracy, stigma and, patients’ lack of phone making contact difficult	Improved HIV and Maternal, Neonatal and Child Health (MNCH) service delivery.
Cole 2018 [55]	Mozambique	CFIR	Mixed methods	IR used to guide post-implementation evaluation of a maternal health programme.	Health providers in facilities involved in implementation; Patients benefiting from intervention	**Facilitators**: Programme adaptability, shared perceives and collective goals among stakeholders. **Barriers:** use of volunteer-based implementation with actors outside of the formal health system, with limited retention..	To explore the contextual factors that may have contributed to observed increases in skilled birth attendance.
Cooke 2019 [56]	Tanzania	CFIR	Qualitative	IR-oriented implementation and evaluation of an integrated anti-retroviral therapy and opioid treatment programme.	Patients benefiting from intervention; Health providers in facilities involved in implementation	**Facilitators**: Clearly understood roles among stakeholders and programme adaptability **Barriers:** lack of space for patient confidentiality and stigma	To understand the contextual factors that influence the effectiveness of integrated methadone and anti-retroviral therapy implementation
Eboreime 2018 [57]	Nigeria	QIF	Qualitative	IR used to guide post-implementation evaluation of a primary health care system improvement intervention.	Primary health care programme managers	**Facilitators:** Adequate pre-intervention planning and stakeholder engagement **Barriers:** Inadequate stakeholder engagements and poor fidelity to planned implementation processes	To identify factors influencing the implementation of a primary health care quality improvement programme.
Eboreime 2019 [58]	Nigeria	MUSIQ	Mixed methods	IR used to guide post-implementation evaluation of a primary health care system improvement intervention.	Primary health care programme managers	**Facilitators:** Subnational political will **Barriers:** suboptimal subnational government leadership, management, financial and technical support	To identify factors influencing a primary health care quality improvement programme’s implementation outcomes
English 2013 [59]	Kenya	CFIR and TDF	Qualitative	IR-oriented pre-implementation evaluation of a child health programme.	Organisations involved in implementation	**Facilitators**: peer pressure, clear communications, provision of feedback, development of a learning climate, leadership engagement, enhancement of self-efficacy **Barriers:** knowledge and skills of personnel	To develop a system-oriented intervention to improve services for children in district hospitals
Finocchario-Kessler 2015 [60]	Kenya	Reach, Effectiveness, Adoption, Implementation, and Maintenance (RE-AIM) model	Quantitative	Using programme data to guide post-implementation evaluation and contextualising lessons learnt.	Mother-infant pairs utilising EID services and government health care providers and lab personnel	**Facilitators**: Strong and sustained collaborations with stakeholders improve the quality and reach of eHealth public health interventions. **Barriers:** Local leadership and resource constraints.	Improve HIV early infant diagnosis (EID)
Gimbel 2016 [61]	Multi-country, including Mozambique, Kenya, Cote d’Ivoire	CFIR	Qualitative	IR-oriented post-implementation evaluation of a mother-to-child-transmission (PMTCT) and paediatric HIV programme	Organisations involved in implementation	**Facilitators**: communication, available resources, external change agents, executing, and reflecting and evaluating **Barriers:** Resistance from the lead nurse	To define the core and adaptable components of a facility-based intervention to address implementation challenges in prevention of mother to child transmission (PMTCT), and identify contextual influences that explain implementation heterogeneity
Gimbel 2017 [62]	Multi-country, including Mozambique, Rwanda, and Zambia	CFIR	Mixed methods	IR-oriented post-implementation evaluation of data quality assessment and improvement activities within the PHIT programmes	Organisations involved in implementation	**Facilitators**: intervention components that aligned with user priorities and government systems and the use of evidence to justify intervention to stakeholders. **Barriers:** Lack of support from District Health Office	To improve aaccess to comprehensive, accurate data to guide resource allocation and programmatic improvement efforts
Jones 2018 [63]	Zambia	CFIR	Mixed methods	IR-oriented mid-implementation evaluation of a voluntary male medical circumcision programme	Lay counsellors and nursing staff	**Facilitators**: Performance evaluation with remedial feedback	Increased acceptability and uptake of voluntary male medical circumcision
Maruma 2018 [64]	South Africa	TFA	Mixed methods	IR-oriented post-implementation evaluation of data collection by community health workers for tuberculosis contact tracing	CHWs	**Facilitators:** Feedback through pre-and post-assessmentsg. **Barriers:** Inadequate training, lack of community acceptance and resource constraints.	To determine factors influencing the collection of information by community health workers for tuberculosis contact tracing
McRobie 2017 [65]	Uganda	CFIR	Mixed methods	IR-oriented pre-implementation evaluation of HIV testing, care and treatment policy implementation	Health providers in facilities involved in implementation	**Facilitators**: donor investment and support, strong scientific evidence, low policy complexity, phased implementation and effective planning. **Barriers:** Limited human resources, infrastructure and health management information systems.	To assess implementation of national HIV policies regarding testing, treatment, and retention at health facilities serving two health and demographic surveillance sites
Nabyonga-Orem 2014 [66]	Uganda	(MRT)	Mixed methods	IR-oriented post-implementation evaluation of barriers and facilitating factors to the uptake of evidence in the process of user fee abolition	Donors, policymakers, researchers, civil society, journalist, private service provider	**Facilitators**: The political window and alignment of the evidence with overall government discourse enhanced uptake of evidence	Uptake of evidence in the process of user fee abolition
Naidoo 2018 [67]	South Africa	CFIR	Qualitative	IR-oriented post-implementation evaluation of community-based HIV programmes	Community members, CHWs, team leaders, facility staff, community leaders, and social workers	**Facilitators**: networking and peer-support, recognition of the CHWs by the government, standardised training. **Barriers:** Limited space and infrastructure for CHWs to work in.	To explore barriers and facilitators to implementation of community-based HIV programmes in order to produce actionable findings to improve them
Newman-Owiredu 2017 [68]	Multi-country, including Malawi, Nigeria, and Zimbabwe	NR	Mixed methods	IR-oriented post-implementation review of HIV (PMTCT) implementation capacity building activities	Health care workers, research team members, and community members support staff (Expert Mothers/Mother Mentors/Mother Support Groups)	**Facilitators**: financial incentives offered as part of national training exercises. **Barriers:** health workers’ perception of research as additional work rather than an opportunity to learn or develop professionally.	Scaling up Option B+ antiretroviral treatment and retention in care.
Petersen Williams 2015 [69]	South Africa	CFIR	Qualitative	Use of IR for pre-implementation design of a screening, referral and treatment programme for substance use among women receiving antenatal care	Health providers in facilities involved in implementation	**Facilitators**: training, adequate support, guidance, and mentoring. **Barriers:** intervention being considered as additional work, lack of interest from staff, time constraints, staff shortages, overburdened workloads, and language barriers.	To investigate health care providers’ perceptions of the acceptability and feasibility of providing screening, brief intervention, and referral to treatment to address maternal substance use among pregnant women attending antenatal care
Rodriguez 2017 [70]	South Africa	CFIR	Qualitative	Qualitative interviews, focus groups discussions, workshop with district directors, clinic leaders, staff, and patients. Prospective programme evaluation.	Health providers in facilities involved in implementation; health policy and health system leaders at national or subnational levels; patients benefiting from intervention	**Facilitators:** Leader support, and employee readiness and motivation **Barriers:** Hierarchical relationships between staff	To identify barriers and facilitators in the implementation, uptake, and sustainability of PMTCT protocols in a rural areas
Rodriguez 2019 [71]	Zambia	CFIR	Mixed-methods	Quantitative and qualitative evaluations of organisational, burnout, and organisational readiness functioning and barriers to implementation.	Health care providers	**Facilitators:** community engagement, leadership support, employee readiness and motivation. **Barriers:** Resource constraints and poor communication of programme benefits.	Uptake of voluntary medical male circumcision
Soi 2018 [72]	Mozambique	CFIR	Qualitative	IR-oriented post-implementation evaluation of the scale-up of an human papillomavirus (HPV) vaccination programmes	Health providers and educators in facilities and schools involved in implementation; health and education policy and health system leaders at national or subnational levels	**Facilitators**: Health workers’ beliefs in importance of vaccines and an organisational culture of making personal sacrifice for immunisation, advocacy and social mobilisation through the right opinion leaders and champions **Barriers:** weak infrastructural characteristics and insufficient organisational incentives	To identify implementation barriers and facilitators affecting the scale-up of HPV vaccination in Mozambique
Warren 2017 [73]	Kenya	CFIR	Qualitative	Using IR an analytical lens for post-implementation evaluation of a complex, multifaceted maternal health programme	Community, facility (nurses and midwives), and policy stakeholders (ministry of health), Federation of Women’s Lawyers - FIDA	**Facilitators**: individual champions, Collaboration with civil-society organisations like FIDA	To address the causes of mistreatment during childbirth and promote respectful maternity care
White 2019 [74]	Benin	CFIR	Mixed methods	IR used to guide mid- implementation evaluation of a quality improvement programme as well as the contextualisation of evaluation findings.	Health providers in facilities involved in implementation	**Facilitators**: Surgical enthusiasm, self-efficacy and motivation, in-depth stakeholder engagement at multiple levels **Barriers:** Staff viewed the checklist as irrelevant or a waste of time and leadership appeared arrogant.	To measure the sustainability of surgical safety checklist use and to evaluate the acceptability, adoption, appropriateness, feasibility and fidelity of nationwide checklist implementation, including penetration of the checklist into operating room culture
Zitti 2019 [75]	Mali	CFIR	Qualitative	IR used to guide post-implementation evaluation of a pilot performance based healthcare financing programme	Health facility staff and managers	**Facilitators:** Implementing a pilot project and good leadership	Gaining contextual understanding of performance-based healthcare financing.

*CFR* Consolidated Framework for Implementation Research, *CHW* Community health workers, *IR* Implementation research, *NR* Not reported, *MRT* Middle range theory, *MUSIQ* Model for Understanding Success in Quality, *NPT* Normalization Process Theory, *QIF* Quality implementation framework, *RE-AIM* Reach Effectiveness Adoption Implementation Maintenance, *TDF* Theoretical Domains Framework, *TFA* Theoretical framework for acceptability, *UHC* Universal health coverage

**Fig. 3:**
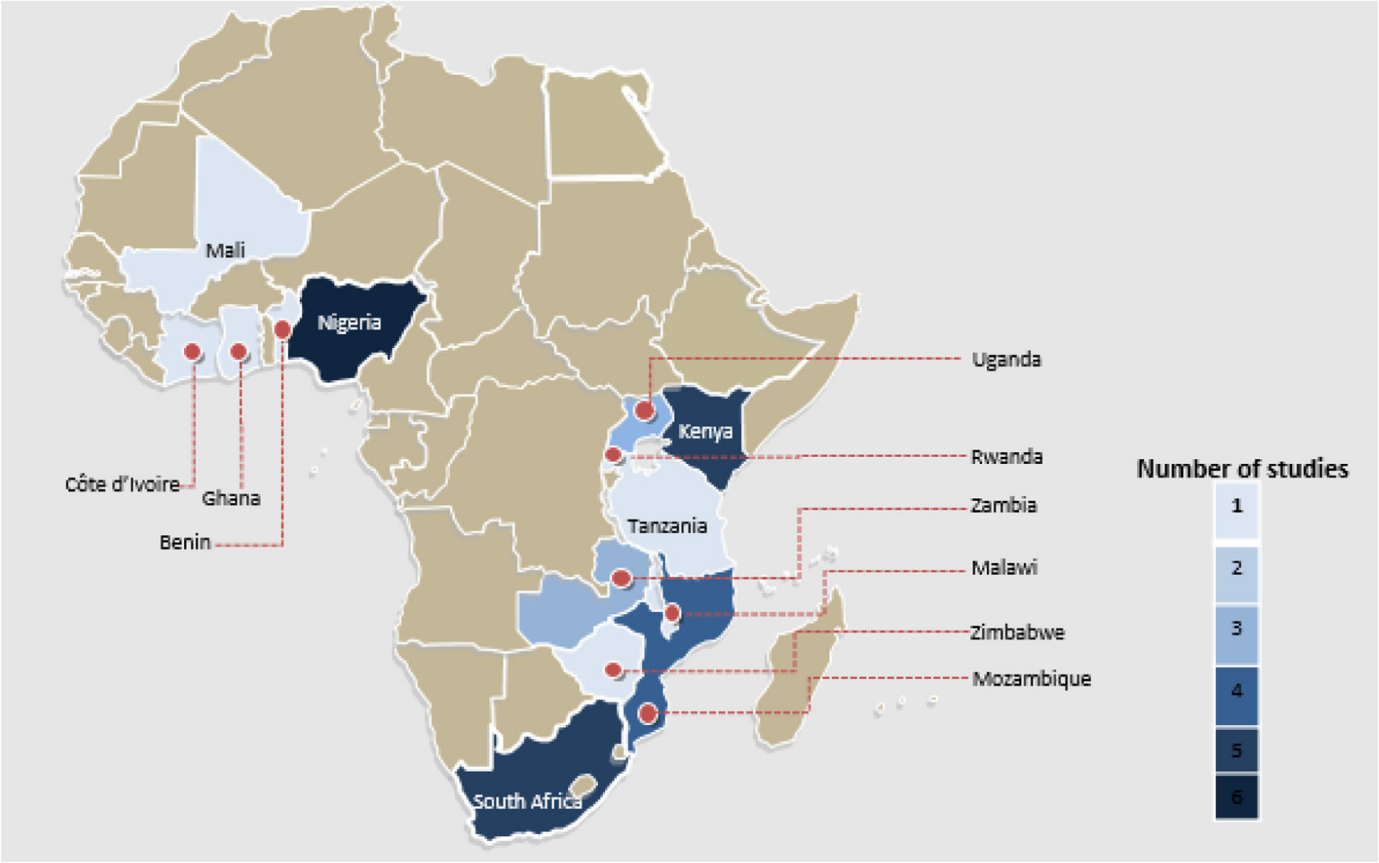
Geographical representation of included studies

The studies’ publication years ranged from 2013 to 2019. The articles addressed implementation research and UHC in 14 different African countries, including Nigeria (*n* = 6), Kenya (*n* = 5), South Africa (*n* = 5), Mozambique (*n* = 4), Zambia (*n* = 3), Uganda (*n* = 2), Benin (*n* = 1), Côte d’Ivoire (*n* = 1), Ghana (*n* = 1), Malawi (*n* = 1), Mali (*n* = 1), Rwanda (*n* = 1), Tanzania (*n* = 1) and Zimbabwe (*n* = 1). Five of the included studies were conducted in multi-country contexts. See Fig. 3.

There were ten distinct UHC-related themes of focus across the articles, including HIV (*n* = 8) [54, 56, 60, 61, 65, 67, 68, 70], maternal and child health (*n* = 4) [55, 59, 69, 73], immunisation (*n* = 3) [50, 51, 72], voluntary male medical circumcision (*n* = 2) [63, 71], healthcare financing (*n* = 2) [66, 75], healthcare data quality (*n* = 2) [62, 64], primary health care quality improvement (*n* = 2) [57, 58], malaria diagnosis (*n* = 1) [52], surgery (*n* = 1) [74] and typhoid fever (*n* = 1) [53].

Most studies addressed all three dimensions of UHC Cube, with the majority involving the use of implementation research to improve access to health services and the quality of health care. Only two studies particularly addressed the healthcare financing dimension [66, 75].

Qualitative study designs (*n* = 12) and mixed methods (*n* = 12) were the most commonly used implementation research study designs, while only two studies used solely quantitative designs. Common qualitative methods utilised across the studies included focus group discussions and key informant interviews. Mixed methods studies used a combination of these with analysis of quantitative data from routine health facility and programmatic records and health worker questionnaire surveys. The consolidated framework for implementation research (CFIR) was the most commonly used implementation research framework (*n* = 17). Other frameworks used included the Mid-Range Theoretical framework (*n* = 1), Model for Understanding Success in Quality (*n* = 1), Normalisation Process Theory (*n* = 1), Quality implementation framework (*n* = 1), Reach Effectiveness Adoption Implementation Maintenance framework (*n* = 1), Theoretical Domains Framework (*n* = 1) and the Theoretical framework for acceptability (*n* = 1). One study used both CFIR and the Theoretical Domains Framework (TDF), while another did not report the specific implementation research framework used.

Broadly, implementation research was used to guide the design, implementation and evaluation of health programmes and services as well as the contextualisation of evaluation findings to improve future implementation outcomes. Implementation research was most frequently used for post-implementation evaluation of implemented health programmes or activities [51, 53, 55, 56, 61, 62, 66–68, 70–73, 75]. Four studies used implementation research approaches for pre-implementation assessment, piloting and planning [50, 59, 65, 69]; while four studies involved mid-implementation evaluation [54, 60, 63, 74]. Specific implementation research activities included the use of implementation research frameworks or theories to guide planning, stakeholder engagement, data collection, data analysis and implementation evaluation, as well as the identification of implementation facilitators and barriers. The majority of the studies (*n* = 16) targeted health care workers. Others targeted and policymakers and health system leaders at national or subnational levels (5 studies), community or lay health workers (*n* = 3), non-profit organisations implementing health programmes (2 studies) and patients or individuals seeking health services (*n* = 2). Eleven of the studies focused on more than one category of target participants.

The implementation research framework analytical domains, themes and constructs used varied across studies. Of the studies that used the CFIR, most reported at least one domain or theme used with their corresponding constructs. The most commonly evaluated domains were intervention characteristics, outer setting, inner setting, characteristics of individuals/teams, process and outcomes domains. Complexity and networks and communication were the mostly commonly used constructs. The study by Finocchario-Kessler and colleagues used the RE-AIM model and reported findings in the implementation reach, effectiveness, adoption, implementation, and maintenance domains [60]. Nabyonga-Orem and colleagues used the MDT to guide content thematic analysis and reporting of findings [66].

The most commonly reported contextual facilitators were political support for programme funding and implementation [51, 53, 55, 56, 61, 62, 66–68, 70–73, 75]; strong and sustained collaborations among stakeholders [59, 65, 69]; goal sharing among stakeholders [51, 55], effective leadership and administrative support for frontline personnel, peer-support [73] and staff motivation [74, 75]. Others included intervention flexibility [51, 55], health workers’ confidence in the intervention [51]; supportive leadership [59, 70]; implementing a pilot project before the actual implementation [75]; and performance appraisal with remedial feedback [59, 63]. The most commonly reported barriers included inadequate human resource and other health care resource and infrastructural gaps, lack of leadership [74], lack of incentives, perception of implementation as additional work burden by personnel [74] and socio-cultural barriers. Others included the hierarchical relationships between staff [70], use of volunteer-based implementation with actors outside of the formal health system with limited retention and adverse hierarchical relationships and tension among healthcare providers [55].

As the purpose of the scoping review was to aggregate evidence and present a summary of the evidence rather than to evaluate the quality of the individual evidence, a formal quality appraisal of included literature was not undertaken.

## Discussion

This review identified 26 studies that utilised implementation research to address UHC-related issues, ranging from specific diseases to performance-based financing and evidence-based decision-making. This suggests a rapid growth in the use of implementation research to promote UHC-related outcomes in the African region. Consistent with the findings of previous reviews, our review shows that qualitative methods were the most commonly applied methodological design, followed by mixed methods, while quantitative methods were the least commonly used [76, 77]. The increasing use of qualitative methods in implementation research has been driven by the suitability of qualitative enquiry for eliciting the perspectives of implementation stakeholders and gaining a deeper understanding of the implementation context. Although, none of the studies explicitly used implementation research in the context of UHC, the implementation outcomes reported in the included studies all related to at least one dimension of UHC Cube, with the majority of them aiming to improve access to health services and quality of health care, while a few particularly addressed the healthcare financing dimension.

This review also found that implementation research can be applied at multiple stages of implementation; before, during and after implementation. Pre-implementation application can be used to prospectively assess organisational readiness and potential implementation barriers or facilitators, which are important for informing UHC policy making, programme design, planning and implementation. At the implementation level, implementation research can be used to monitor implementation progress, track utilisation of resources and identify implementation gaps. At the post-implementation stage, implementation research can be used to evaluate what worked (effectiveness and facilitators) and what did not work (failures and implementation barriers), as well as to interpret and contextualise those findings.

Specific implementation research activities depended on study design. Qualitative methods were mostly used to guide the development of semi-structured interview guides or focus group protocols, data collection and the development of qualitative coding templates for analysis. On the other hand, quantitative methods often involved the use of implementation research frameworks to guide survey question development and quantitative data analysis. Mixed-method approaches used both qualitative and quantitative methods complementarily. While qualitative interviews of implementation stakeholders were the most common implementation research activities, specific activities depended on the stage of implementation in which they were conducted. For example, McRobie and colleagues applied the CFIR framework to guide pre-implementation baseline data collection and analysis, and identify potential enablers and barriers to the implementation of national HIV policies regarding testing, treatment and retention in Uganda [65]. In Zambia, Jones and colleagues used a mid-implementation design to identify and analyse predictors of a voluntary male medical circumcision programme’s success or failure to create an ‘early warning’ system that enables remedial action during implementation [63]. Naidoo and colleagues used a post-implementation CFIR to map contextual barriers and facilitators to the implementation of community-based HIV programmes in order to produce actionable findings to improve them within the South African context [67].

The diversity of target participants and UHC-related contexts across studies in this review reflects the multi-dimensional, multi-stakeholder and multi-level utilisation of implementation research studies to promote UHC-related outcomes. It also reflects the adaptation of implementation research approaches to take into account the complexity of the health systems within the study settings. Another important finding of this review is that the CFIR was the most commonly used implementation research framework, which may reflect its compatibility for use in African context as previously noted by Means and colleagues in their review [76].

The most commonly reported contextual facilitators such as political support, sustained funding, supportive institutional leadership, financial incentives, clarity of goals as well as strong collaboration among stakeholders, are consistent with those reported in previous reviews [76, 78]. Conversely, the most frequently reported barriers, such as insufficient funding, inadequate human resource and other health care resource and infrastructural gaps, lack of incentives, perception of implementation as additional work burden and socio-cultural barriers have also been previously reported [76]. It is also evident that weak political commitment poses a major implementation barrier [74]. Given its economic costs and social implications, the UHC agenda is intensely political, with contested scope and diverse stakeholders capable of facilitating or hindering its progress [29, 79]. As such, advancing the use of implementation research in the context of UHC will require strong political support for; funding, mobilisation of stakeholders and the uptake of the evidence generated to inform UHC-oriented policy making, governance, implementation and monitoring. It is imperative to take these facilitators and barriers into account when designing contextually appropriate implementation research strategies for promoting the attainment of UHC-related goals.

This review highlights the growing interests in the use of implementation research and data-driven decision making for improving health outcomes at the population level in African contexts. However, the further uptake of implementation research is constrained by numerous barriers as earlier outlined, in addition to the scarcity of good quality, consistently available, complete and reliable health data, particularly at facility and health programme levels [80]. Implementation research presents a practical opportunity for investment in routine health service and programme data collection to improve the quality and availability of essential health services. Overall, the lessons learnt from the various ways in which implementation research has been applied can help to inform future efforts at planning, implementing and tracking the performance of health programmes in achieving UHC-related outcomes; improving service delivery, increasing population coverage and facilitating wider access, while fostering health system strengthening and resilience in the African region.

One way of increasing the uptake and use of implementation research is by leveraging existing monitoring and evaluation systems which have already been substantially established across African countries. However, integrating implementation research into conventional monitoring and evaluation systems will require addressing challenges resulting from sometimes onerous donor reporting requirements. Donor-driven data reporting requirements often result in duplicate reporting systems, burdening the limited human resources at health facility and programme levels [62]. In addition, human resource gaps and inadequate research capacity, knowledge and skills which often constrain the conduct of implementation, need to be addressed. Failure to address the human resource shortages can lead to situations such as health workers’ perception of implementation research activities as additional work rather than an opportunity to learn and improve health service outcomes, as reported by many of the studies included in this review. Thus, personnel recruitment, regular training, guidance, and mentoring are all essential for successful implementation research activities, as are efforts to address the structural divide between policy makers, implementers and researchers.

### Limitations

As with any scoping review, our review is not without limitations. While the search strategy was designed to be sensitive enough to capture relevant literature, it may still have missed some. To minimise this, we reviewed the search term iteratively to incorporate related terminologies as we became more familiar with the literature and performed manual review of references. Although we searched a relevant grey literature database, it is difficult to comprehensively search for and locate these sources of evidence and some may have been missed. To ensure feasibility of the review, only one reviewer (CAN) screened, selected and extracted all the data. However, every step of these processes, as well as the extracted data were reviewed and verified by the review team. Another important limitation of this review is that, as in most scoping reviews; a formal quality appraisal of included literature was not undertaken. As such, the strength of the evidence cannot be ascertained. While our literature search was comprehensive, covering both peer-reviewed and relevant grey literature; it is possible that the review did not include all relevant literature available, as some may not have been accessible at the time of literature search. It is also important to acknowledge that the included studies did not directly or explicitly aim to assess UHC outcomes of implementation research. All outcomes reported were however well related, albeit implicitly, to at least one of the three UHC dimensions.

## Conclusion

While still limited, there is a body of evidence on the use of implementation research in the attainment of UHC-related outcomes in Africa, including the improvement of routine data for decision-making, efficient resource allocation, as well as the improvement of the availability, accessibility, affordability and quality of health services. Therefore, there is a need for more attention and investment in this type of research. This review has also identified important facilitators and barriers to the use of implementation research in UHC-related contexts in the African region, which need to be considered when designing future implementation research strategies.

## Supplementary Information

**Additional file 1.** PubMed/MEDLINE search strategy.

## Data Availability

All data analysed in this review are available in the included published articles.\

## Abbreviations

AHPSR: Alliance for Health Policy and Systems Research
CFIR: Consolidated Framework for Implementation Research
CHW: Community Health Workers
EIDM: Evidence-informed Decision Making
MRT: Middle range theory
MUSIQ: Model for Understanding Success in Quality
NPT: Normalization Process Theory
PRESS: Peer Review of Electronic Search Strategies
PRISMA-ScR: Preferred Reporting Items for Systematic Reviews and Meta-Analyses Extension for Scoping Reviews
QIF: Quality implementation framework
RE-AIM: Reach Effectiveness Adoption Implementation Maintenance
TDF: Theoretical Domains Framework
UHC: Universal Health Coverage
