# Supplementary material for: Implementation research approaches to promoting universal health coverage in Africa: a scoping review
Source: BMC Health Serv Res. 2021 May 3;21:414. doi: 10.1186/s12913-021-06449-6 (PMC8094606; doi:10.1186/s12913-021-06449-6)
Supplement: Supplementary file 1 — Additional file 1. PubMed/MEDLINE search strategy. [file 12913_2021_6449_MOESM1_ESM.docx]

**Appendix 1:** PubMed/MEDLINE search strategy

| **Search #** | **Search Texts and Syntaxes** |
| --- | --- |
| #1 | "implementation science"[Title/Abstract] OR "implementation research"[Title/Abstract] OR "decision science"[Title/Abstract] OR "decision research"[Title/Abstract] OR "improvement science"[Title/Abstract] OR "improvement research"[Title/Abstract] |
| #2 | "knowledge translation"[Title/Abstract] OR "knowledge management"[Title/Abstract] OR "dissemination science"[Title/Abstract] OR "dissemination research"[Title/Abstract] |
| #3 | "evidence-based medicine"[MeSH Terms] OR "evidence based medicine"[Title/Abstract] OR "evidence based healthcare"[Title/Abstract] OR "evidence based health care"[Title/Abstract] OR "evidence informed decision making"[Title/Abstract] |
| #4 | #1 OR #2 OR #3 |
| #5 | “Universal health coverage”[Title/Abstract] OR "health equity”[Title/Abstract] OR Health[Title/Abstract] OR “health access”[Title/Abstract] OR “financial risk protection"[Title/Abstract] OR “health access"[Title/Abstract] OR access[Title/Abstract] OR equity[Title/Abstract] |
| #6 | Africa OR African OR Algeria OR Angola OR Benin OR Botswana OR Burkina Faso OR Burundi OR Cameroon OR “Canary Islands” OR “Cape Verde” OR “Central African Republic” OR Chad OR Comoros OR Congo OR “Democratic Republic of Congo” OR Djibouti OR Egypt OR Eritrea OR Eswatini OR Ethiopia OR Gabon OR Gambia OR Ghana OR Guinea OR “Ivory Coast” OR “Cote d'Ivoire” OR Jamahiriya OR Kenya OR Lesotho OR Liberia OR Libya OR Madagascar OR Malawi OR Mali OR Mauritania OR Mauritius OR Mayotte OR Morocco OR Mozambique OR Namibia OR Niger OR Nigeria OR Principe OR Reunion OR Rwanda OR “Sao Tome” OR Senegal OR Seychelles OR “Sierra Leone” OR Somalia OR “St Helena” OR “sub-Saharan Africa” OR Sudan OR Swaziland OR Tanzania OR Togo OR Tunisia OR Uganda OR “Western Sahara” OR Zaire OR Zambia OR Zimbabwe |
| #7 | #4 AND #5 AND #6 |
